# Evaluation of Three Mutations in Codon 385 of Glucose-6-Phosphate Dehydrogenase via Biochemical and In Silico Analysis

**DOI:** 10.3390/ijms252312556

**Published:** 2024-11-22

**Authors:** Adriana Gálvez-Ramírez, Abigail González-Valdez, Beatriz Hernández-Ochoa, Luis Miguel Canseco-Ávila, Alexander López-Roblero, Roberto Arreguin-Espinosa, Verónica Pérez de la Cruz, Elizabeth Hernández-Urzua, Noemi Cárdenas-Rodríguez, Sergio Enríquez-Flores, Ignacio De la Mora-De la Mora, Abraham Vidal-Limon, Saúl Gómez-Manzo

**Affiliations:** 1 Laboratorio de Bioquímica Genética, Instituto Nacional de Pediatría, Secretaría de Salud, Mexico City 04530, Mexico; galvezramirezadriana@gmail.com; 2 Departamento de Biología Molecular y Biotecnología, Instituto de Investigaciones Biomédicas, Universidad Nacional Autónoma de México, Mexico City 04510, Mexico; abigaila@biomedicas.unam.mx; 3 Laboratorio de Inmunoquímica, Hospital Infantil de México Federico Gómez, Secretaría de Salud, Mexico City 06720, Mexico; beatrizhb_16@ciencias.unam.mx; 4 Facultad de Ciencias Químicas, Campus IV, Universidad Autónoma de Chiapas, Tapachula City 30580, Mexico; cansecoavila@gmail.com (L.M.C.-Á.); usdalexander@hotmail.com (A.L.-R.); 5 Departamento de Química de Biomacromoléculas, Instituto de Química, Universidad Nacional Autónoma de México, Mexico City 04510, Mexico; arrespin@unam.mx; 6 Neurobiochemistry and Behavior Laboratory, National Institute of Neurology and Neurosurgery “Manuel Velasco Suárez”, Mexico City 14269, Mexico; veped@yahoo.com.mx; 7 Laboratorio de Toxicología Genética, Instituto Nacional de Pediatría, Secretaría de Salud, Mexico City 04530, Mexico; elyzabet91@yahoo.com.mx; 8 Laboratorio de Neurociencias, Instituto Nacional de Pediatría, Secretaría de Salud, Mexico City 04530, Mexico; noemicr2001@yahoo.com.mx; 9 Laboratorio de Biomoléculas y Salud Infantil, Instituto Nacional de Pediatría, Secretaría de Salud, Mexico City 04530, Mexico; sergioenriquez@ciencias.unam.mx (S.E.-F.); ignaciodelamora@ciencias.unam.mx (I.D.l.M.-D.l.M.); 10 Red de Estudios Moleculares Avanzados, Clúster Científico y Tecnológico BioMimic, Instituto de Ecología A.C. (INECOL), Carretera Antigua a Coatepec 351, El Haya, Xalapa 91073, Mexico

**Keywords:** mutations, G6PD, molecular dynamics simulations

## Abstract

Glucose-6-phosphate dehydrogenase (G6PD) deficiency is an enzymopathy that affects approximately 500 million people worldwide. A great number of mutations in the *G6PD* gene have been described. However, three class A G6PD variants known as G6PD Tomah (C385R), G6PD Kangnam (C385G), and G6PD Madrid (C385W) have been reported to be clinically important due to their associations with severe clinical manifestations such as hemolytic anemia. Therefore, this work aimed to perform, for the first time, biochemical and functional characterizations of these variants. The G6PD variants were cloned and purified for this purpose, followed by analyses of their kinetic parameters and thermal stability, as well as in silico studies. The results showed that the mutations induced changes in the proteins. Regarding the kinetic parameters, it was observed that the three variants showed lower affinities for G6P and NADP^+^, as well as lower thermal stability compared to WT-G6PD. Molecular dynamics simulations showed that C385 mutations induced changes around neighboring amino acids. Metadynamics simulations showed that most remarkable changes account for the binding pocket volumes, particularly in the structural NADP^+^ binding site, with a concomitant loss of affinity for catalytic processes.

## 1. Introduction

The cytosolic enzyme glucose-6-phosphate dehydrogenase (G6PD) is found in both prokaryotic and eukaryotic cells [1,2,3,4]. In humans, the *G6PD* gene is positioned neighboring the telomeric region of the X chromosome. The *G6PD* gene encodes a specific protein of 515 amino acids and a molecular mass of 59 kDa. G6PD protein plays a regulatory role in the pentose–phosphate pathway (PPP), a highly conserved metabolic pathway that produces a variety of critical molecules, for example, nucleotide precursors and nicotinamide adenine dinucleotide phosphate reduced (NADPH). G6PD catalyzes the first reaction of the oxidative phase of the PPP, allowing for the reduction of NADP^+^ at the expense of the dehydrogenation of glucose-6-phosphate (G6P) to 6-phosphogluconolactone (6PGL) and generating NADPH [5].

In erythrocytes, the PPP is the only route to generate reduced forms of NADPH as they do not contain mitochondria. Therefore, G6PD activity has a crucial role in protecting cells from oxidative injury due to increased reactive oxygen species [6,7]. In addition, NADPH plays an important role in redox homeostasis, which is involved in the reduction of glutathione, and glutathione is the primary protection against reactive oxygen species and helps avoid red cell hemolysis [8,9,10].

A whole of 230 mutations distributed throughout the *G6PD* gene have been reported, including single-point mutations, causing single amino acid substitutions and multiple mutations (two or more substitutions) [11,12,13]. These mutations lead to decreased activity and stability of the G6PD enzyme, triggering oxidative injury in RBCs and a variety of clinical symptoms known as G6PD deficiency [4,8]. G6PD deficiency is an enzymopathy that affects approximately 500 million people worldwide (7.5% of the world’s population). This enzymopathy can be symptomless or can generate severe clinical symptoms such as chronic hemolytic anemia (G6PD variant class A), neonatal jaundice and acute hemolytic anemia generated by some drugs, fava beans, or infection (G6PD variant class B), and no hemolysis (G6PD variant class C) [13].

**Figure 1:**
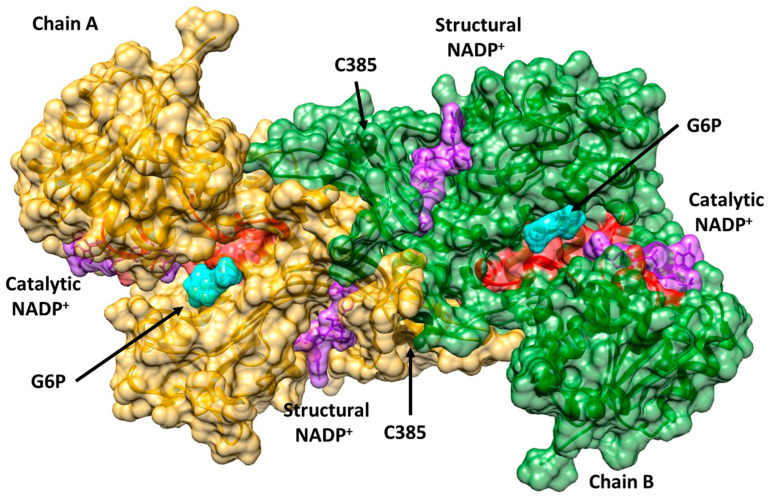
The structure of the human glucose-6-phosphate dehydrogenase dimer (G6PD; PDB entries 2BH9 and 2BHL). The locations of the class A mutations Tomah (C385R), Kangnam (C385G), and Madrid (C385W) are indicated in chain B (green chain and black spheres), while in chain A (yellow chain), the Cys residue is marked as a black sphere. Glucose-6-phosphate (G6P) is drawn in cyan, while catalytic nicotinamide adenine dinucleotide phosphate (NADP^+^) and structural NADP^+^ are drawn in purple.

In the mutant G6PD Tomah, a transition of T → C in nucleotide 1153 was identified, which resulted in a Cys → Arg substitution at position 385 [14]. This mutation was identified in two unrelated Spanish G6PD-deficient males with chronic nonspherocytic hemolytic anemia (CNSHA) located in exon 10. On the other hand, in a genetic description of Korean patients diagnosed with glucose-6-phosphate dehydrogenase deficiency, the mutant G6PD Kangnam was identified. The patient in which the Kangnam mutant was identified was a 5-year-old boy who had fever and paleness. The patient visited the hospital after recuperating from a critical clinical condition. The patient presented a G6PD enzyme level of 2.1 U/g Hb, and both the Coombs and the osmotic fragility tests were negative. The G6PD deficiency was categorized as class I, corresponding to the World Health Organization (WHO) classification, due to the drastic decreased G6PD enzyme activity and development of CNSHA. The child had no family genetic history of hematological disorders and had a new T > G mutation at position 1153, which gave rise to a Cys → Gly amino acid replacement at position 385 (Figure 1). According to the WHO, this variant belongs to G6PD class I (now variant class A) [15]. Finally, the mutation G6PD Madrid was identified in two Spanish males with G6PD deficiency and hemolytic anemia. One of the patients was diagnosed at birth since he showed signs of neonatal jaundice. At 6 years old, the child still showed moderate to severe CNSHA. G6PD Madrid variant showed undetectable residual activity in RBC. A transition of C to G was identified at nucleotide 1155, causing a Cys → Trp replacement in the amino acid residue at position 385. According to the WHO classification, this mutation corresponds to the G6PD variant class A [16]. These G6PD mutations in exon 10 result in variants related to neonatal jaundice and life-long CNSHA [17]. These reports highlight the significance of exon 10 of the *G6PD* gene in the stabilization of the G6PD enzyme.

It is important to emphasize that in G6PD deficiency, various mutations in the same codon have been reported where the native amino acid residue was changed by different amino acid residues, producing different G6PD variants that have been reported in different countries around the world. In this sense, three mutations in codon 385, located in exon 10, have been reported. They give rise to the class A G6PD variants (previously class I variants), G6PD Tomah (C385R), G6PD Kangnam (C385G), and G6PD Madrid (C385W) (Figure 1). All these variants are related to clinical manifestations such as chronic hemolytic anemia. In addition, it is essential to mention that these mutations are distant from the active site and the catalytic NADP^+^ region; however, they are near the “structural” NADP^+^ site.

Based on the above, in this work, we report the cloning, purification, thermostability assays, and detailed steady-state kinetics for the three clinical variants G6PD Tomah, Kangnam, and Madrid for the first time, which were registered in the same codon (385) and identified in various parts of the worldwide. In addition, based on the three-dimensional (3D) structure of the G6PD protein and using in silico approaches to mine structural information on the effects of the G6PD variants, we identified modifications in the protein structure to understand the clinical symptoms of these mutations in order to explain the molecular mechanisms observed in patients with G6PD deficiency.

## 2. Results and Discussion

### 2.1. Construction and Expression of Recombinant G6PD Variants

Three clinical single mutants, G6PD Tomah (C385R), Kangnam (C385G), and Madrid (C385W), were obtained via site-directed mutagenesis. The mutation into the *G6PD* gene was confirmed via DNA sequence in the generated plasmids. As shown in Supplementary Figure S1A, a single nucleotide change of thymine to cytosine (T → C) at nt 1153 was observed (Figure S1A). In electropherograms for the single mutant G6PD Kangnam, a change of thymine for guanine (T → G) at nt 1153 was observed (Figure S1B). In addition, for G6PD Madrid, the nucleotide guanine was replaced by thymine (G→T) at nucleotide (nt) 1154 (Figure S1C). Therefore, we confirmed the desired mutations in each of the variants that were obtained.

### 2.2. Purification of Recombinant G6PD Variants

To analyze the effects of the mutations on the activity and native structure of the wild-type (WT-G6PD) enzyme, recombinant WT-G6PD, and the three G6PD variants were purified via the anion-exchange chromatography (Q-Sepharose) method and affinity (2′5′ADP Sepharose 4B) methods [4,5,12,18,19,20,21]. It was observed that lower protein concentrations were obtained from 2 L of culture for each variant compared to WT-G6PD (4.8 mg/mL). The variant with the highest protein concentration was G6PD Tomah (0.8 mg/mL), followed by the Madrid variant (0.57 mg/mL) and the Kangnam variant (0.23 mg/mL). It is important to highlight that although the three variants purified in this study occurred at the same structural position (amino acid residue 385), different total protein concentrations were obtained for each of them. One of the factors involved in this low protein expression was probably the physicochemical properties of the residues involved in each of the variants (Arg, Gly, and Trp), which have different characteristics than the Cys in the WT-G6PD. These results are in agreement with the report by our working group, in which the concentrations of recombinant proteins were lower than the WT-G6PD enzyme [4,5,12,18,19,20,21], likely suggesting poor folding in the native structures of these variants or a loss of stability.

### 2.3. Kinetic Analysis

Kinetic analysis was performed to determine the effects of the three mutations in the WT-G6PD on the Michaelis–Menten affinity constant (K_m_) for the physiological substrates (G6PD and NADP^+^) and the catalytic constant (*k*_cat_). As seen in Supplementary Figure S2, for the WT-G6PD, a K_m_ of 38.4 and 6.1 µM were obtained for the G6P and NADP^+^ substrates, respectively, with a *k*_cat_ of 223 s^−1^ (Table 1). Subsequently, as shown in Figure S2B, the variant G6PD Tomah showed a K_m_ of 64.9 µM for the G6P substrate and a K_m_ of 13.8 µM for the NADP^+^ substrate (Figure S2E), with a *k*_cat_ of 15 s^−1^. Figure S2C shows kinetic plots for the variant G6PD Kangnam, where a K_m_ of 55.2 µM for the G6P substrate was obtained. Similarly, in Figure S2G, a K_m_ of 10.3 µM for the NADP^+^ substrate was calculated, with a *k*_cat_ of 0.4 s^−1^. Finally, the variant G6PD Madrid showed a K_m_ of 78.2 µM and a K_m_ of 12.2 µM for the G6P and NADP^+^ substrates, respectively (Figure S2D,H), with a *k*_cat_ of 40.5 s^−1^ (Table 1). For the three class A G6PD variants analyzed in this study, their affinities for the physiological substrates (NADP^+^ and G6P) decreased regarding native G6PD, and the catalytic constant (*k*_cat_) was also affected by the mutations. The three mutants analyzed in this study presented reductions in catalysis (*k*_cat_), suggesting that the catalytic site was affected. The variant G6PD Kangnam was the most affected, with a catalysis loss of 99% regarding the WT-G6PD. The variant G6PD Tomah had 91% less activity, followed by the variant G6PD Madrid, which showed a loss of activity of 76% regarding the WT-G6PD. These results suggest that the G6PD variants had reduced affinities for physiological substrates and showed reductions in catalysis, suggesting that this loss of activity was closely related to the clinical manifestations of CNSHA found in each of the patients where these mutations were identified.

Similar results were obtained for the class A G6PD variants Zacatecas, Durham, Veracruz, Nashville, Guadalajara, and Yucatán, which showed greater affinity losses for their substrates regarding class II and III variants. Specifically, it is interesting to mention that the positions of the mutations of the three variants G6PD Tomah (C385R), Kangnam (C385G), and Madrid (C385W) are located near the position of the G6PD Guadalajara variant (R387C, class A), where the mutation occurs near the structural NADP^+^ binding site and dimer interface. The results of this work are similar to the report by Martínez-Rosas et al. [20] for the variant G6PD Guadalajara. In addition, it should be noted that the three mutants of interest in this study are not located near the catalytic binding sites of NADP^+^ and G6P; however, the affinities for the substrate and the catalytic binding site were affected.

### 2.4. Thermal Inactivation Analysis

**Figure 2:**
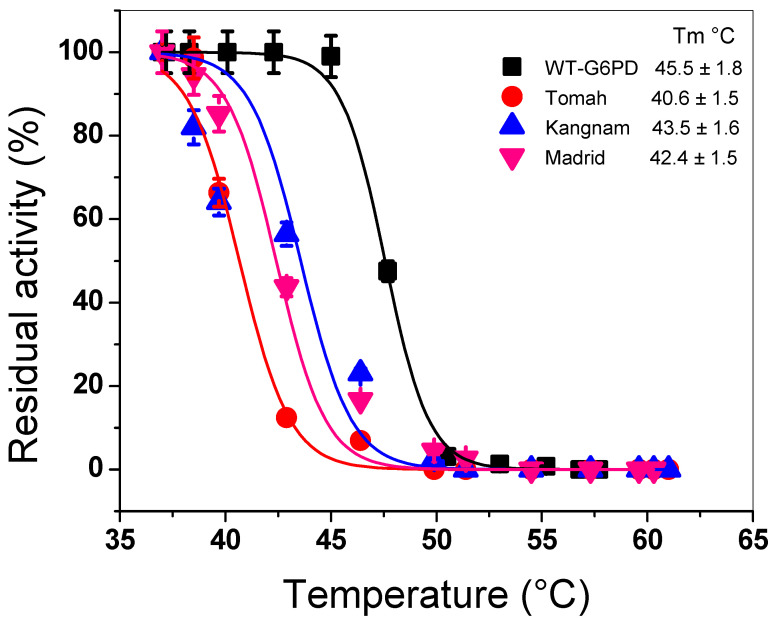
Thermal inactivation trials of three clinical mutants and WT-G6PD. The recombinant proteins were suspended at 0.2 mg/mL and incubated at 37 to 60 °C for 20 min. The residual activity was calculated and expressed as a percentage concerning their controls. The trials were performed in triplicate, with standard errors (SEs) ± <5%.

To evaluate the effects of the mutations on the stability of the native protein, thermal stability trials were carried out for WT-G6PD and the variants. This assay has been widely used as an indicator of alterations in protein stability [4,5,10,12,18,19,20,21,22,23]. The residual activities of each of the variants were plotted at different temperatures, and it was observed that the catalytic activity decreased as the temperature increased (Figure 2). For the variant G6PD Tomah, a T_50_ value of 40.6 °C was observed, while for the variants G6PD Kangnam and G6PD Madrid, T_50_ values of 43.5 °C and 42.4 °C were obtained. These T_50_ values were 4 to 7 °C lower regarding the WT G6PD, which showed a T_50_ value of 47.5 °C. These results suggest that the mutations in codon 385 lead to a more unstable G6PD protein. In addition, it was identified that the Tomah variant presented the greatest instability, with a 7 °C decrease in the T_50_ value, followed by the variants Kangnam and Madrid. These results are in agreement with the obtained for the G6PD variant Guadalajara (R387C), where a T_50_ value of 43.3 °C was obtained. The mutations analyzed in this study were located near the structural NADP^+^ binding site and dimer interface in the 3D structure of the native G6PD protein, which is involved in dimer formation and enzyme stability, as observed in the class A G6PD variants Nashville, Fukaya, Campinan, Wisconsin, Durham, Guadalajara, and Mount Sinai [18,20,23,24]. Therefore, it was proposed that G6PD instability could be a frequent effect of these deleterious mutations. It is noteworthy that all results showed that the variants analyzed in this study presented reductions in catalytic activity and alterations in their stability, suggesting that these two alterations are responsible for the clinical manifestations in patients with G6PD deficiency.

### 2.5. In Silico Analysis

**Figure 3:**
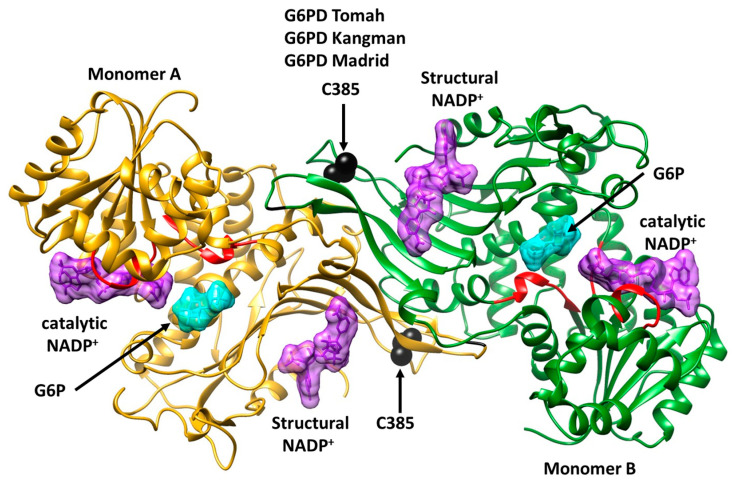
Structure of the WT-G6PD dimer (PDB ID: 2BH9 and 2BHL). The position of the class A G6PD variants Tomah, Kangnam, and Madrid is identified at codon 385 by substituting the amino acid cysteine (black spheres). Catalytic G6P, NADP^+^, and structural NADP^+^ are shown in light blue and purple. Monomers A and B are shown in bright yellow and green ribbons.

To evaluate the effects of the mutations on the WT-G6PD protein, we performed in silico mutagenesis. Figure 3 shows the mutations in the protein structure. It can be observed that the amino acid C385 is located near the structural NADP^+^ binding site, which is important for dimer formation and promotes the stability of the protein [25,26,27]. As it is a crucial site, any amino acid change at this position could lead to a reduction in the structural binding of the NADP^+^ molecule and could prevent the generation of the active form of the protein (i.e., dimerization), leading to the CNSHA pathogenicity associated with G6PD deficiency. With the in silico mutations, we set out to explain and understand the influences of the mutations on the structures of the Tomah, Kangnam, and Madrid variants, as well as the causes of their severity, using bioinformatics methods and biochemical trials of these variants was not entirely possible due to difficulties in obtaining enough pure proteins.

**Figure 4:**
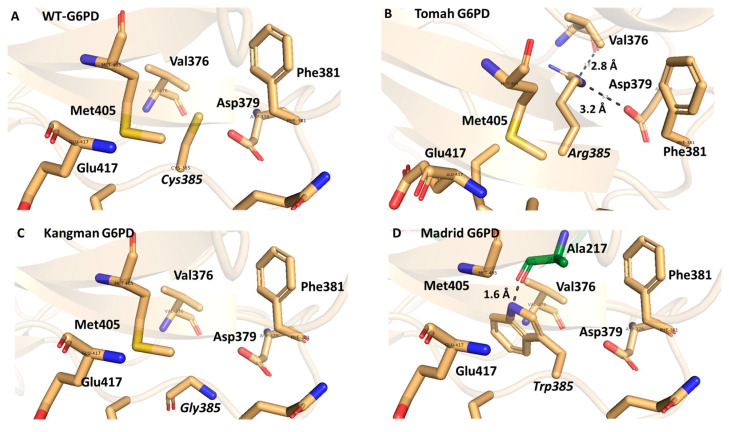
Structural comparison of WT-G6PD (PDB ID: 2BH9 and 2BHL) and minimized G6PD variants class A Tomah, Kangman, and Madrid models. (**A**) WT-G6PD. (**B**) In silico mutation to generate the Tomah variant. (**C**) In silico mutation to generate the Kangman variant. (**D**) In silico mutation to generate the Madrid variant. Interactions are shown as black dotted lines.

The Tomah variant of G6PD (C385R) is caused by a change in a nitrogenous base from thymine to cytosine at nucleotide 1153, which leads to the amino acid cysteine being substituted with arginine. Cys is a polar, uncharged amino acid with a molecular weight (MW) of 121 Da (Figure 4A). When it is mutated into Arg, it becomes a polar, positively charged amino acid with a MW of 174 Da. A bulky guanidino-group sidechain occupies the space, forming close interactions with V376 and D379 in chain A (Figure 4B). G6PD Kangnam (C385G) is a single mutation provoked by the substitution of guanine for thymine (1153 T → G), which leads to a change from cysteine to glycine, a small nonpolar amino acid with a MW of 75 Da, whose sidechain is a H atom, which probably causes the area to become more flexible and consequently causes the protein to lose stability (Figure 4C). On the other hand, G6PD Madrid (C385W) includes the replacement of cytosine with guanine at nucleotide 1155, which leads to a change from cysteine to tryptophan, whose residue is apolar and hydrophobic, with an indole ring in its sidechain and a molecular weight of 204 Da. The bulky indole sidechain closely interacts with A217 in chain B (Figure 4D).

Generating in silico mutations allowed us to analyze the interactions between amino acid 385 and neighboring amino acids. The amino acids Arg, Gly, and Trp present in each variant have side chains with different sizes, shapes, polarities, charges, and hydrophobicities about Cys, an amino acid in WT-G6PD. This causes changes in the environment around position 385 and throughout the protein, which probably leads to losses of stability and catalytic efficiency and, consequently, the clinical manifestations of these variants. As interactions between amino acids help the protein to fold and fluctuate to adopt the active structure, any amino acid modification can cause structural alterations that impact the intermolecular interactions in the protein structure.

### 2.6. Use of In Silico Approaches to Mine Structural Information on the Effects of the G6PD Variants

Bioinformatics approaches have revolutionized the field of molecular biology, enabling researchers to conduct detailed structural analyses of enzymes such as G6PD at the molecular level. Determining the impacts of mutations (insertions, deletions, or missense variations) in protein structures on stability and activity is one of the most exciting areas in protein sciences. In the case of G6PD, extensive studies have differentiated pathogenic variants and determined how these mutations impact overall stability [11,28]. Outstanding efforts were made by several groups working on in silico approximations to predict protein stability from sequences [6,29,30,31]. For example, different stability predictors have been developed, including some outstanding tools that distinguish between pathogenic variants when combining artificial intelligence (machine learning) or evolutionary information [32]. However, as there is an enormous sequence–annotation gap between the available sequences, which continues to grow, there are gaps, such as the sequence–structure gap, that need to be mined to access more detailed diagnoses, public policies, or even personalized medicine approaches. The most accurate stability predictors for pathogenic mutations are structure-based methods, which require available protein structures [33,34].

With the advent of sequence–structure methodologies based on artificial intelligence such as AlphaFold (AF2) [35] (https://alphafold.ebi.ac.uk, accessed on 1 February 2024), a wide range of problems are being tackled in structural biochemistry. Due to the immediate impacts of these artificial intelligence systems developed by DeepMind, several groups sought to understand the protein stability problem. For example, Pak et al. [36] explored the capacity for AF2 predictions to evaluate the impacts of mutations on protein stability changes (ΔΔG) despite the software having a disclaimer stating that the routine “*has not been validated for predicting the effect of mutations*” (https://alphafold.ebi.ac.uk/faq, accessed on 1 February 2024). Using data on experimentally measured effects of mutations on protein stability, their analysis indicated that overall, AF2 metrics can estimate ΔΔG values; however, the effects of mutations on *pLDDT* scores (a superposition-free metric that indicates the extent to which a protein model reproduces a reference structure) were weakly correlated with ΔΔG and protein function. The formulation of a metric to serve as a predictor of the impacts of mutations on protein stability and function is part of the ongoing effort to map the effect of every G6PD mutation.

On the other hand, Keskin et al. [37] reported other examples of the effects of mutations on protein stability using AI methodologies and structural analysis. The overall performances of five protein stability predictors were evaluated, namely, mCSM, MAESTRO, CUPSAT, SAAF2EC-SEQ, and MUpro. This research group also used AF2-computed structures of 26 hereditary cancer-associated proteins to analyze a breast cancer cohort of 355 patients. The results showed that the pathogenicity labels of the missense mutations in the cohorts had an unbalanced distribution. Despite the stability predictors showing moderate performances when discriminating pathogenic variants, an improvement was obtained from an AF2 structure set with high pathogenicity prediction power. These findings suggest that using protein stability predictors in combination with structure-based metrics can aid in predicting the pathogenicity of missense mutations [37]. Further research is needed to improve the performance of stability predictors, as well as to assess changes in the internal dynamics of mutant proteins, such as changes in hydrogen bond patterns, changes in solvation energies, and thermodynamics changes in entropic terms associated with structurally modified protein scaffolds, among others. This type of prospective approach can be applied to the G6PD mutant library as different metrics can be enriched with structural effects of mutations close to the binding site.

Several approximations of G6PD variants’ effects on protein structure and stability have been reported. A fine-tuned structural characterization of the presence of structural NADP^+^ in G6PD dimers was performed by Horikoshi et al. [38]. Despite the large amount of clinical information about these mutations, the atomic and molecular mechanisms underlying the losses of G6PD enzymatic activity and stability due to the absence of structural NADP^+^ and the role of dimerization in catalysis remained unclear. To gain insight into these mechanisms, the researchers determined the structures of several class A G6PD variants: F381L, R393H, V394L, and P396L. They aim to propose a molecular mechanism for the loss of catalytic activity and stability using full structural characterization (X-ray crystallography, cryogenic electron microscopy (cryo-EM), small-angle X-ray scattering (SAXS), and biophysical analyses). Comparisons of the mutants to the WT-G6PD enzyme through molecular dynamics simulations (MDSs) also exposed a mechanism for severe G6PD deficiencies due to the class A G6PD variants. The mechanism suggests that mutations around the NADP^+^ binding site alter the b-strand dynamics of dimerization, resulting in inactive scaffolds.

The variants p.(C385R) or Tomah, p.(C385G) or Kangman, and p.(C385W) or Madrid were classified as pathogenic alleles (evidence categories PS3, PM1, PM2, PM5, PP2, PP3, and PP4) according to proposed by the American College of Medical Genetics and Genomics and the Association for Molecular Pathology (ACMG/AMP) [39] (https://www.medschool.umaryland.edu/Genetic_Variant_Interpretation_Tool1.html/, accessed on 1 February 2024). In silico analyses of predictions were performed using PolyPhen (http://genetics.bwh.harvard.edu/pph2/, accessed on 1 February 2024) [40], MutPRed (http://mutpred.mutdb.org/, accessed on 1 February 2024) [31], and Align GVGD (http://agvgd.hci.utah.edu/agvgd_input.php, accessed on 1 February 2024) [41,42] programs. Polyphen classified the Tomah and Kangman variants as benign. In contrast, the Madrid variant was classified as likely damaging. MutPred indicated that the variants had a loss of the allosteric site at N388, and Align GVGD indicated that the variants had altered function (Table S1). The results obtained in the present work through in vivo functional studies and in silico predictions support the damaging effect on the protein.

#### 2.6.1. Molecular Dynamics Calculations of Dimeric G6PD Variants

**Figure 5:**
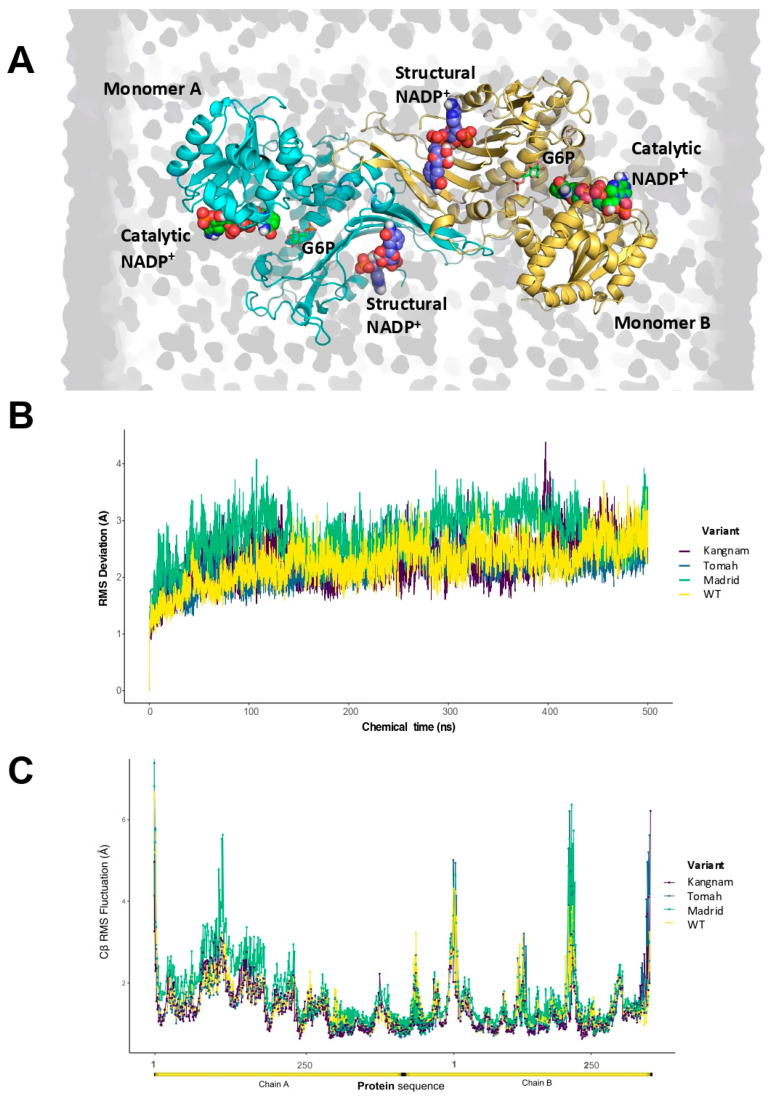
Explicit all-atom molecular dynamics simulations of G6PD variants complexes. (**A**) Evaluated dimeric G6PD model system in explicit solvent. (**B**) Alpha carbon (Cα) RMSD calculations of the G6PD variants. Calculated average RMSD values were 2.3 Å for WT, 2.5 Å for Tomah, 2.7 Å for Kangnam, and 3.0 Å for the Madrid variant. The values were averaged for ±5 aa from N- and C-terminal groups. (**C**) Beta carbon (Cβ) RMSF calculations (sidechains fluctuations) of the G6PD variants. The shown fluctuations were averaged for 500 ns of MDSs.

Different molecular simulation techniques on dimeric G6PD can help reveal numerous limitations posed by docking trials, such as rotamer selection, cofactor charge description, and orientation inside the binding site. In this context, explicit solvent molecular dynamics simulations (MDSs) can sample sidechain orientations and free rotations of cofactors. Moreover, MDSs can improve the sampling of the binding process since solvent waters are explicitly described, resulting in a refining structural outcome with all-atom positions being calculated. These evaluations help to visualize the possible conformational changes associated with C385 point mutations. We aimed to perform conventional MDS simulations to evaluate potential changes in ligand-binding affinity for structural and catalytic NADP^+^ and the glucose-6-phosphate (G6P) substrate. During the MDS calculations, the G6PD complexes were first relaxed and subsequently clustered with a hierarchical analysis, which assessed highly existent conformations of G6PD dimers and ligands complexes (Figure 5A). The root mean square deviation (RMSD) for stereogenic centers or alpha carbons and root mean square fluctuations for side chains or beta carbons (RMSFs) were monitored. The dominant conformations of the C385 point mutations in complexes with NADP^+^ and G6P are depicted in Figure 5B.

**Figure 6:**
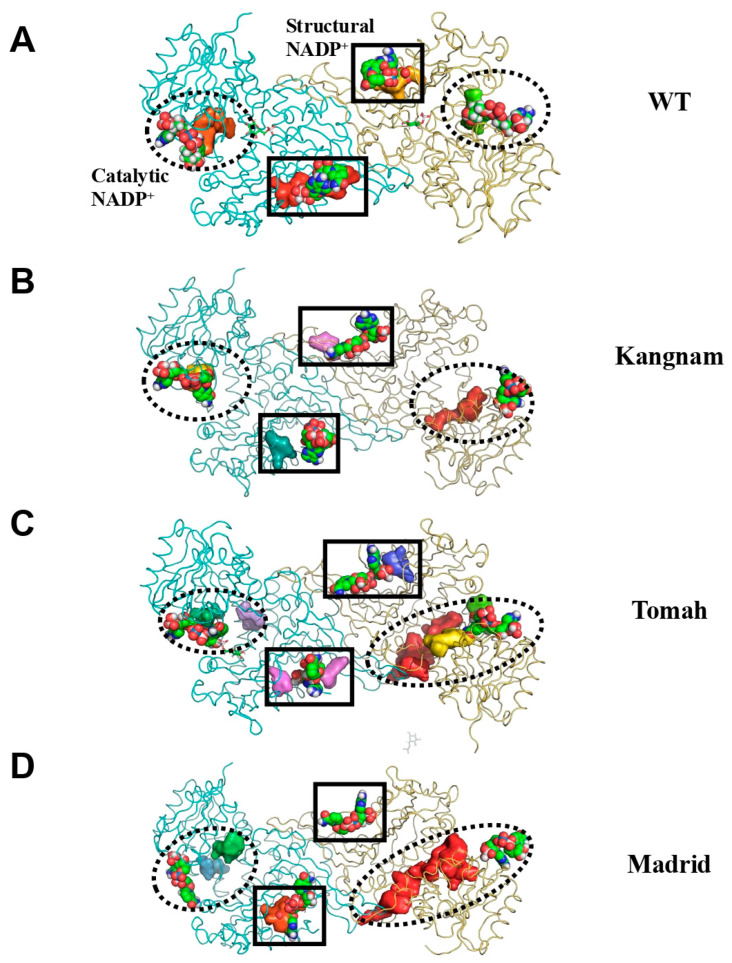
The average changes in the NADP^+^ binding pockets of the G6PD variants: (**A**) G6PD WT, (**B**) Kangnam, (**C**) Tomah, and (**D**) Madrid. The identified binding pockets are shown as solid surfaces. Protein structures are shown as green ribbons, and NAPD^+^ is shown as licorice sticks. Binding pockets for NADP^+^ are identified by circles for catalytic NADP^+^ and squares for structural NADP^+^.

Introducing an explicit solvent and long chemical simulation times to the complexes slightly refined them, with an average RMSD of cc. 3.0 Å. Severe structural changes could be discarded for overall quaternary dimer structures, as conformational changes were not detected in any simulations despite the point mutations. Moreover, the RMS fluctuations showed that for the Tomah (C385R) variant, the sidechain flexibility increased by cα. 2 Å, in contrast to the Madrid (C385W) and Kangnam (C385G) variants, which showed reduced flexibility in the N-terminal and β-strand domains close to the structural NADP^+^ (Figure 5C). However, the most remarkable changes were recorded in the volumes of the binding pockets during all chemical simulations, as depicted in Figure 6A–D, where single-point mutations could create relevant changes in the NADP^+^ environment.

Table 2 shows the NADP^+^ binding pocket volume changes due to the C385 single-point mutations. The most remarkable changes were found in the Madrid (C385W) variant, which had aromatic and bulky sidechains that could reduce the available volume around the structural NADP^+^ binding site. This variant showed an increase in the volume of the structural and catalytic NADP^+^ binding sites of ca. 10 Å^3^, implying a fine restructuring of the binding sites that was not perceptible through RMSD calculations.

Overall, the changes in the volumes of the NADP^+^ binding sites were remarkable; however, the quaternary structure was not affected in any dimeric complex, suggesting a combinatorial effect resulting in differences in the apparent affinities for ligands and the formation of the dimeric complex (Figure S3) [43].

#### 2.6.2. Evaluation of Binding and Unbinding of G6PD Dimer Assembly with Metadynamics Simulations

**Figure 7:**
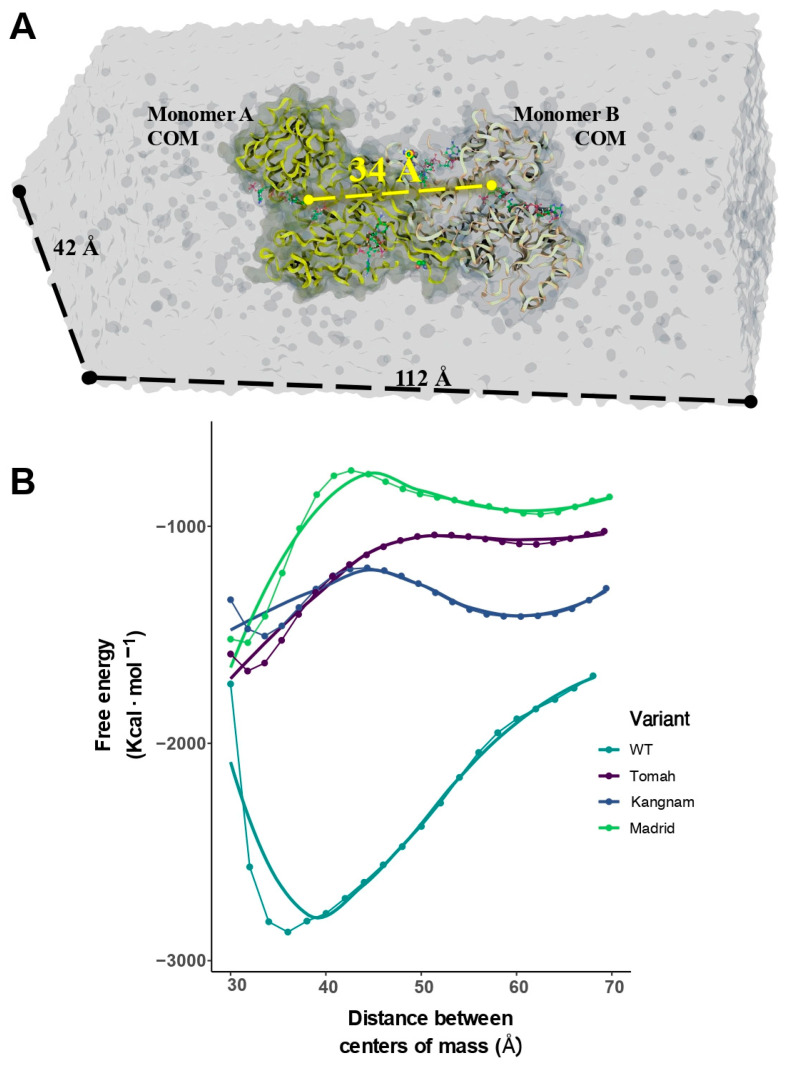
A metadynamics-solvated model system and a collective variable description of the evolution of the bound/unbound states of the G6PD variants. (**A**) The configuration of the metadynamics-enlarged simulation box. (**B**) The potential mean forces of the dimerizations of the G6DP variants. All depicted values were averaged from triplicate calculations for each variant.

A well-tempered metadynamics methodology was enhanced with a sampling technique for the binding and unbinding process of two monomers for each G6PD variant to evaluate the possible role of the point mutations in forming the active dimeric complex. The potential mean force (PMF) change of the dimeric assembly was averaged from three independent simulation trajectories, where all previously simulated conditions were reproduced, resulting in PMF as a descriptor of quaternary structure stability. In the metadynamics simulations, the maximal value of external force over the center of mass was described as a rupture force (unbinding), and the pulling work (binding) averaged from all trajectories was evaluated over 34 Å of intermonomer distance or a collective variable (CV) (Figure 7A).

Like a ligand-binding process, pulling work can be considered more valuable than the rupture force because it can be associated with free energy via Jarzynski’s isobaric–isothermal average, such as in steered molecular dynamics methods [44]. The average pulling forces of the G6PD monomers were time-dependent for an additional 100 ns (Figure 7B). The results indicated that the mean rupture forces for WT-G6PD ranged from −2800 Kcal·mol^−1^ at intermonomer distances of 30–40 Å and during the binding process to −1800 Kcal·mol^−1^ when the dimer was fully dissociated in the simulation box. The data suggested that dimer formation was a very stable process with the most negative values when the center-of-mass (COM) distance reached cα. 35 Å; however, the monomers tended to dissociate in the presence of a significant amount of energy, as depicted for higher CV distances.

The Tomah (C385R) and Kangnam (C385G) variants displayed similar PMF profiles compared to WT-G6PD (Table 3) but with reduced energetic barriers between processes (−490.94 and −108.08, respectively). These processes were not as stable as the dimerization process of WT-G6PD but could lead to a displacement in the equilibrium that could stabilize the dimeric active form. However, both variants displayed changes in their catalytic NADP^+^ binding sites, indicating that both enzymes lost their apparent affinities for both catalytic processes. The difference between the bound and unbound states displayed changes in the work profiles and was identified as a critical factor for the binding of monomers. According to metadynamics calculations, the average pulling work values of the three tested variants fell within 35.74 Å and ca. −1300 kcal·mol^−1^ (Table 3). Furthermore, the Madrid variant (C385W) was a complex case due partly to a stable dimerization process (∆∆G = −703.77 kcal·mol^−1^) but had the most significant changes in the volumes of both NAPD^+^ binding sites. Fine-tuning the molecular scaffolds can affect the transformation rates of G6P, as it has been reported that small changes in the architectures of redox enzymes can have exponential impacts on electron transfer rates [45].

Finally, within the therapeutic implications, this work contributes that the three G6PD variants analyzed were identified in male patients that, at the moment of the identification, showed CNSHA. Since no functional data were available for Tomah, Kangman, and Madrid G6PD variants, we determined experimentally the principal biochemical properties of these enzymes. Moreover, it is essential to note that, through understanding the implications of genetic variants for enzyme structure, different groups have shown that structural analysis based on molecular dynamics simulations [44] and artificial intelligence techniques [45] can represent a crucial step toward identifying a potential therapeutic approach to correcting G6PD deficiency.

Our biochemical results in this study showed that the alterations caused by these mutations at the functional and structural levels in the native structure of the G6PD protein resulted in a loss of catalytic activity and stability of the variants regarding WT-G6PD. Moreover, it is remarkable because the G6PD deficiency is high where malaria is prevalent [8], and it has been proposed that it inhibits the growth of specific types of malaria, giving rise to protective adaptation. However, tafenoquine was approved in 2018 by the Food and Drug Administration for treating malaria. Tafenoquine eliminates the hypnozoites of *Plasmodium vivax* and has a long half-life, where a single dose of this drug is as effective as an entire 14-day course of primaquine [13]. Moreover, it has been observed that in patients with G6PD deficiency, tafenoquine and primaquine can produce acute hemolytic anemia [46]. Moreover, the set of clinical, molecular, and biochemical data and in silico predictions will allow us to classify a variant as “pathogenic”, “likely pathogenic”, “uncertain significance”, “likely benign”, and “benign”, as recommended by ACMG/AMP. So, when a variant is reported to be pathogenic, healthcare providers are very likely to consider it “applicable” and modify a patient’s treatment or monitoring.

At present, blood transfusions are the only treatment option for this disease, particularly for the most severe class A G6PD variants. This study provides insights that could help identify potential therapeutics to correct G6PD deficiency and improve the search for chemical space to develop suitable small-molecule drugs to control this prevalent disease. This breakthrough could pave the way for a more effective and accessible treatment option for G6PD deficiency, improving the lives of millions of people worldwide.

A limitation of our study is that it was carried out at the molecular and biochemical levels, where, through site-directed mutagenesis, we created the three clinical G6PD variants and carried out the biochemical studies involved in this study. In addition, we related the assays obtained in this study with those observed in patients where the mutation was identified, who presented chronic nonspherocytic hemolytic anemia (CNSHA). However, to have a better explanation of these mutations at a physiological level, in the future, studies in animal models will have to be carried out, as was previously performed by Rovira et al. [47] that demonstrated the integration of the *hG6PD* gene in totipotent stem cells using a retroviral vector. The authors determined that the expression of hG6PD in vivo was stable and provided a rescue of G6PD deficiency in stem cells. Moreover, a similar vector containing the human *G6PD* gene (VSV-G vector) was adequate for the expression of hG6PD in Macaque monkeys [48].

## 3. Materials and Methods

### 3.1. Construction, Expression, and Purification of Recombinant G6PD Variants

The clinical G6PD mutants Tomah, Kangnam, and Madrid were obtained via site-directed mutagenesis, according to reports by Gómez-Manzo et al. [5]. Mutagenic oligonucleotides were designed for each mutant from the human *G6PD* gene sequence deposited in the Gen Bank database (accession: NM 001042351.2) (Table 4). The polymerase chain reaction (PCR) was reproduced using a previously reported method [4,5,12,18,19,20,21]. The PCR products were analyzed via electrophoresis in 1% agarose gel and visualized using GelRed (Nucleic Acid Gel, Biotium, Fremon, CA, USA). Then, the PCR products were digested with a *Dpn*I enzyme for 2 h at 37 °C. Afterward, the final digestion was used to transform competent *E. coli* TOP-10 cells, which were selected on a solid LB agar plate containing 200 µg/µL carbenicillin (CB-200). Then, the plasmid DNA was purified and verified via sequencing to confirm the desired G6PD mutants (pETgC385R, pETgC385G, and pETgC385W). Finally, it was used to transform into competent *E. coli* BL21(DE3)∆*zwf*::*kan*^r^ cells.

To obtain crude extracts of the G6PD variants Tomah, Kangnam, and Madrid, liquid LB cultures were carried out with CB-200 (200 µg/µL) plus kanamycin (200 µg/µL). The cultures were grown, induced, harvested, and lysed via sonication according to a previously reported method [4,5,12,18,19,20,21]. The sonication product was centrifuged, and the crude extract was obtained. Finally, the enzymatic activity of each of the crude extracts was determined with a standard reaction mixture (100 mM Tris-HCl, 1 mM NADP^+^, 1 mM G6P, and 3 mM MgCl_2_ at a pH of 8.0).

The recombinant G6PD variants were purified using anion-exchange columns (Q-Toyopearl and Q-Sepharose) and affinity columns (2′,5′-ADP Sepharose 4B). First, the crude extract was loaded in an anion-exchange Q-Toyopearl column. Using a linear gradient from 0 to 0.35 M, the proteins were eluted. The portions with specific activity for G6PD were collected and loaded onto the 2′5′ADP Sepharose 4B affinity column. The column was washed until the absorbance at 280 nm was zero. Proteins were eluted from the column with 100 µM NADP^+^ in the equilibrium buffer. The G6PD`s activity was measured, and the fractions with enzymatic activity were concentrated using Amicon YM-30 tubes (Millipore, Bedford, MA, USA). Subsequently, the concentrate was loaded onto the Q-Sepharose 4B column (5 mL), which was previously equilibrated. The column was washed, and the recombinant protein was eluted with a linear gradient of NaCl from 0 to 0.35 M. The portions that presented enzymatic activity were concentrated, and the purity of the G6PD variants was assessed by 12% SDS-PAGE gels stained with Coomassie brilliant blue.

### 3.2. Determination of Steady-State Kinetic Parameters

The kinetic parameters of the variants G6PD Tomah, Kangnam, and Madrid were calculated by evaluating the production of NADPH at 340 nm. To obtain the kinetic parameters, one of the substrates was maintained at a saturating concentration (NADP^+^ or G6P), while the concentration of the second substrate was changed from 2.5 to 200 µM (NADP^+^ or G6P). The reaction started with 800 ng of total protein. The kinetic parameters (K*_m_*, *k*_cat_, and V*_max_*) were obtained from the initial velocity and fit of the Michaelis–Menten equation. *k*_cat_ value was obtained from *V_max_*, taking into the molecular mass (59.25 kDa for monomer).

### 3.3. Thermal Inactivation Analysis

Thermal stability trials were performed to determine the effects of the mutations in the WT-G6PD. The G6PD proteins were suspended at 0.2 mg/mL and incubated at temperatures ranging from 37 to 60 °C by 20 min. The residual G6PD activity was determined using a standard reaction mixture. At the ends of the assays, the residual activities were expressed as percentages concerning their controls. The 100% activity was fixed from the enzymes incubated at 37 °C. The experiments were carried out in triplicate.

### 3.4. Analysis of In Silico and Site-Directed Mutagenesis

In order to present the main structural elements of the G6PD enzyme, the three-dimensional structure of the G6PD dimer was constructed with the PyMOL software package (v. 2.5.7) (New York, NY, USA) using files deposited in the Protein Data Bank (1QK1, 2BHL, and 2BH9). Subsequently, the mutation was constructed on the G6PD structure using the mutagenesis wizard to generate the G6PD Tomah (C385R), G6PD Kangnam (C385G), and G6PD Madrid (C385W) variants.

### 3.5. Enhanced Molecular Dynamics Calculations

The enhanced molecular dynamics simulations (MDSs) technique was performed for the three G6PD variants in complex with glucose-6-phosphate and NADP^+^ under *pmemd.cuda* [29] within the Amber20 package (https://ambermd.org/, accessed on 1 April 2023) [29,30]. The GAFF and Amberff14SB parameter sets [31,32] were applied to model protein and substrate organic molecules. The TIP3P explicit water model was applied to simulate ca. 112,780 solvent. The final dimensions of the orthorhombic water box were 155 × 155 × 155 Å for all systems at a pH of 6.8 with 0.15 M NaCl.

The MDS protocol included a minimization routine of 5000 steps under the steepest descendent minimization algorithm, with subsequent minimization under the conjugated gradient algorithm for 50,000 steps. A second simulation stage under simulated annealing protocol and NvT ensemble was performed. All systems were heated with a linear interpolation scheme for 0.3 ns at 10 K, followed by 1 ns at 100 K, 1 ns at 300 K, and 0.3 ns at 400 K. Finally, the systems were cooled down at 303 K for 2 ns. For pre-equilibration, all systems were simulated under the NpT ensemble during 50 ns and equilibrated for an extra 100 ns of conventional molecular dynamics simulations at 1 atm of constant pressure under the control of Monte Carlo Barostat = 1. The temperature of the systems was controlled with an external bath coupling at 303 K. For calculation of the long-range electrostatic interactions, the Particle Mesh Ewald (PME) method was applied (1 × 10^−10^ of tolerance) with an integration step of 2 fs for all periodic systems. Gaussian Accelerated Molecular Dynamics simulations encompass different sampling techniques that smooth potential energy surfaces between biomolecular processes and reduce energy barriers between two or more processes [33,34], such as conformational changes, the binding and unbinding of substrates, and peptide dissociations, among others. A dual boost method was applied to add Gaussian harmonic boost potentials to dihedral angles and potential energy for all calculated systems. However, more in-depth descriptions of this methodology have been published elsewhere [35,36]. The GaMD simulations were performed in triplicate for 500 ns for each G6PD system with sigma0P = 6.0 and sigma0D = 6.0 at 303 K at the same integration time. Each GaMD production replica was combined and analyzed via hierarchical clustering methods with the *cpptraj* v5.0 [37] and VMD v1.9.3 [38] packages to identify conformational changes associated with each mutation. Additionally, the RMSDs of the alpha carbons and the RMSFs of the sidechains were averaged.

Furthermore, as the C385 mutations are allocated around the structural NADP^+^ binding pocket, we suspected that single-point mutations could exert structural changes that modify the substrate affinity. The Python package *Pyvol* [43] was interfaced with PyMOL v. 2.5.7 (New York, NY, USA). It was used to calculate, visualize, and compare the binding pockets volume and SASA in a systematic and reproducible fashion for each G6PD system. *Pyvol* evaluated all the cavities within the dimer models that were accessible to small-radius probes (~2 Å) and stored the information on the identified pockets in the form of sets of coordinates and radii of tangent spheres within a three-dimensional voxel grid. Finally, the volumes of the NADP^+^ pockets were calculated in Å^3^ and SASA in Å^2^.

### 3.6. Metadynamics Simulations

To evaluate the free energy profile of the dimer dissociation, we performed enhanced sampling methods under the well-tempered metadynamics technique [44] since the spontaneous dimerization process is inaccessible to unbiased MDSs due to timescale limitations. This method enhances sampling using collective variables (CVs), such as dihedral rotation, angle or RMSD perturbation, and distance-based stretching; moreover, these variables are steered, akin to an adaptive umbrella sampling theoretical framework [44]. All potential mean forces (Kcal·mol^−1^) were solved for each CV using a histogram of the differentiable function of the Gaussian potential at a specific width and height (ω and σ, respectively). Without a systematic way to choose CVs due in part to the specific conditions of each biomolecular system, the selection of a CV must rely on physically reasonable variables. Our simulations were carried out using Desmond (v.2022-1), and collective variables were based on the distance between the centers of mass for each G6PD monomer, approx. 34 Å, and the stretching along the x-axis. The evaluation provided insight into the effect of each mutation on dimer stabilization and was of significant importance to our study. The Gaussian function parameters for all simulations were set to 0.03 Kcal·mol^−1^ (ω) and 0.5 Å/ps (σ). A rate of 1 ps for Gaussian functions was applied, and a bias factor of 3 was set for the well-tempered algorithm (the rate at which the Gaussian height decreased until reaching a maximum of 70 Å long or full dimer dissociation). Three replicas were generated for each mutation for an additional 100 ns at 1 fs of integration time, and the potential mean force was derived using the Maestro Metadynamics Analysis plugin inside the Maestro 2022-1 suite from Schrödinger ^®^ (New York, NY, USA).

## 4. Conclusions

In this work, we report the cloning, purification, biochemical characterization, and molecular dynamics of three clinically relevant class A G6PD variants—named G6PD Tomah, G6PD Kangnam, and G6PD Madrid—for the first time, which are located in the same codon. The kinetic parameters (K_m_, *V_max_*, and *k_cat_*) of the variants were obtained, and their values were decreased compared to native G6PD, showing impairment in their catalytic activity. The thermal stability of the variant proteins was decreased compared to wild-type G6PD, suggesting that the mutations lead to more unstable G6PD protein. In silico and molecular dynamics analyses of the variants revealed significant structural changes, mainly in the structural NADP^+^ binding site, which could explain the clinical manifestations of G6PD deficiency and open new lines of research to develop new drugs that target these G6PD variants, improving the quality of life of patients.

## Figures and Tables

**Table 1:** The kinetic parameters of WT-G6PD and Tomah, Kangnam, and Madrid variants.

Kinetic Constants	WT-G6PD	Variants		
		Tomah	Kangnam	Madrid
K_m_ G6P (µM)	37.5 ± 1.8	64.9 ± 3.2	66.2 ± 3.1	78.2 ± 3.4
K_m_ NADP^+^ (µM)	7.2 ± 0.3	13.8 ± 0.6	10.3 ± 0.5	12.2 ± 0.5
*k*_cat_ (s^−1^)	223 ± 8.9	15 ± 0.6	0.4 ± 0.01	40.5 ± 1.2
*k*_cat_/K_m_ G6P (s^−1^·M^−1^)	5.9 × 10^6^	0.2 × 10^6^	0.006 × 10^6^	0.52 × 10^6^
*k*_cat_/K_m_ NADP^+^ (s^−1^·M^−1^)	32.3 × 10^6^	1.1 × 10^6^	0.04 × 10^6^	3.3 × 10^6^

The kinetic parameters were obtained from three independent experiments.

**Table 2:** NADP^+^ binding pocket volume changes of G6PD variants.

G6PD Variant	Structural NADP^+^ Binding Pocket Volume (Å^3^)	Catalytic NADP^+^ Binding Pocket Volume (Å^3^)	Structural NADP^+^ SASA (Å^2^)	Catalytic NADP^+^ SASA (Å^2^)
WT	494.3	490.1	261.1	270.9
Tomah	497.4	500.3	289.9	278.2
Kangnam	490.2	491.7	262.2	261.3
Madrid	505.6	507.2	288.3	285.7

All NADP^+^ binding pocket volume and solvent-accessible surface area (SASA) were analyzed with Pyvol v1.0 under PyMOL (Schrödinger, New York, NY, USA) and expressed as the sum of the binding sites from both monomers.

**Table 3:** The free energy profiles of the G6DP enzyme variants for bound/unbound dimerization.

G6PD Variant	ΔG_bound_	ΔG_unbound_	ΔΔG
WT	−2867.57 ± 173.23	−1796.98 ± 122.95	−1070.59
Tomah (C385R)	−1574.32 ± 79.56	−1083.38 ± 101.33	−490.94
Kangnam (C385G)	−1420.67 ± 123.43	−1312.59 ± 102.94	−108.08
Madrid (C385W)	−1556.81 ± 92.30	−853.04 ± 67.90	−703.77

All units were averaged from triplicate metadynamics simulations lasting 100 ns and expressed in kcal mol^−1^.

**Table 4:** The oligonucleotides and strains used to obtain the G6PD mutants Tomah, Kangnam, and Madrid.

Mutant	Mutagenic Oligonucleotide Sequence	Reference
Tomah	Fw: 5′-CACCAGCAGCGCAAGCGCAA-3′	This study
	Rv: 5′-TTGCGCTTGC**G**CTGCTGGTG-3′	
Kangnam	Fw: 5′-CACCAGCAG**G**GCAAGCGCAA-3′	This study
	Rv: 5′-TTGCGCTTGC**G**CTGCTGGTG-3′	
Madrid	Fw: 5′-CACCAGCAGTG**G**AAGCGCAA-3′	This study
	Rv: 5′-TTGCGCTT**C**CACTGCTGGTG-3′	
**Strains**	**Genetic Characteristics**	**Reference**
*E. coli* TOP-10	F^−^, DE(araD-araB)567, lacZ4787(del)::rrnB-3, LAM^−^, rph-1, DE(rhaD-rhaB)568, hsdR514	[49]
*E. coli* BL21(DE3)Δzwf::kan^r^	F^−^ ompT gal dcm lon hsdSB(r^−^ m^−^) λ(DE3 [lacI lacUV5-T7 gene 1 ind1 sam7 nin5]) Δ*zwf-777*::*kan*	[5]

## Data Availability

Data is contained within the article and Supplementary Materials.

## Supplementary Materials

The following supporting information can be downloaded at https://www.mdpi.com/article/10.3390/ijms252312556/s1.
