# Supplementary material for: Evaluation of Three Mutations in Codon 385 of Glucose-6-Phosphate Dehydrogenase via Biochemical and In Silico Analysis
Source: Int J Mol Sci. 2024 Nov 22;25(23):12556. doi: 10.3390/ijms252312556 (PMC11641639; doi:10.3390/ijms252312556)
Supplement: Supplementary file 1 [file ijms-25-12556-s001.zip › ijms-3258712-supplementary.pdf]

Article

Evaluation of three mutations in codon 385 of glucose-6-phosphate dehydrogenase via biochemical and in silico analysis

Adriana Gálvez-Ramírez, Abigail González-Valdez, Beatriz Hernández-Ochoa, Luis Miguel Canseco-Ávila, Alexander López-Roblero, Roberto Arreguin-Espinosa, Verónica Pérez de la Cruz, Elizabeth Hernández-Urzua, Noemi Cárdenas-Rodríguez, Sergio Enríquez-Flores, Ignacio De la Mora-De la Mora, Abraham Vidal-Limón,*, and Saúl Gómez-Manzo,*

A

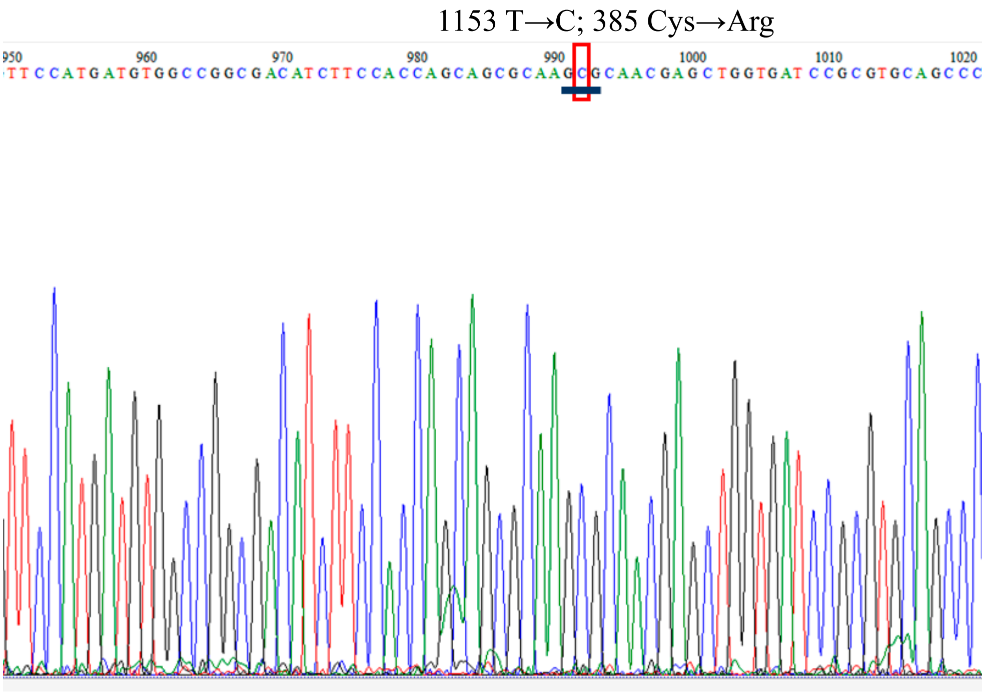

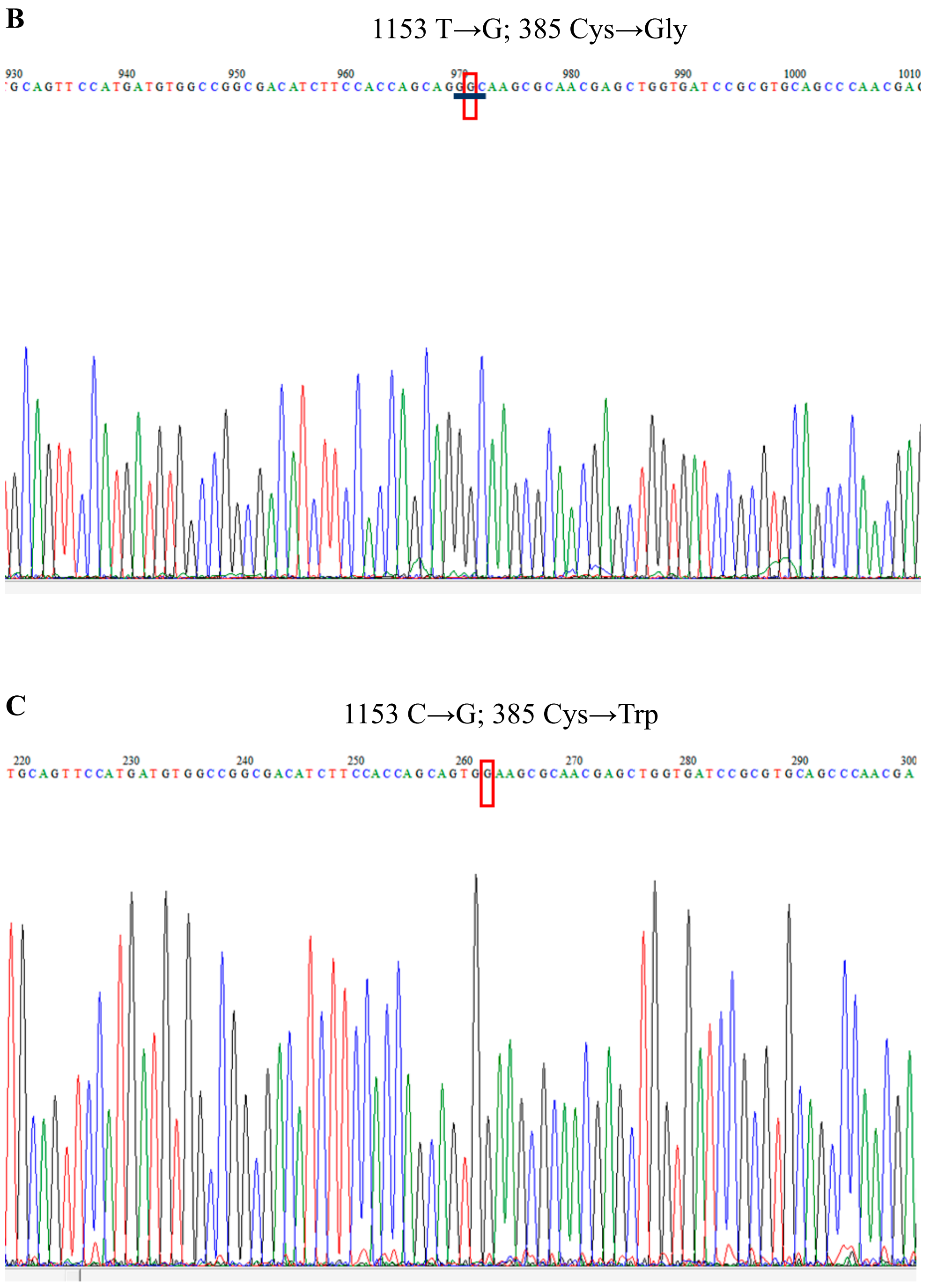

Figure S1. Electropherogram of mutants G6PD in codon 385. (**A**) G6PD Tomah (T1153C, C385R). (**B**) G6PD Kangnam (T1153G, C385G), and (**C**) G6PD Madrid (C1155G, C385W). The location of the mutation is indicated in red boxes.

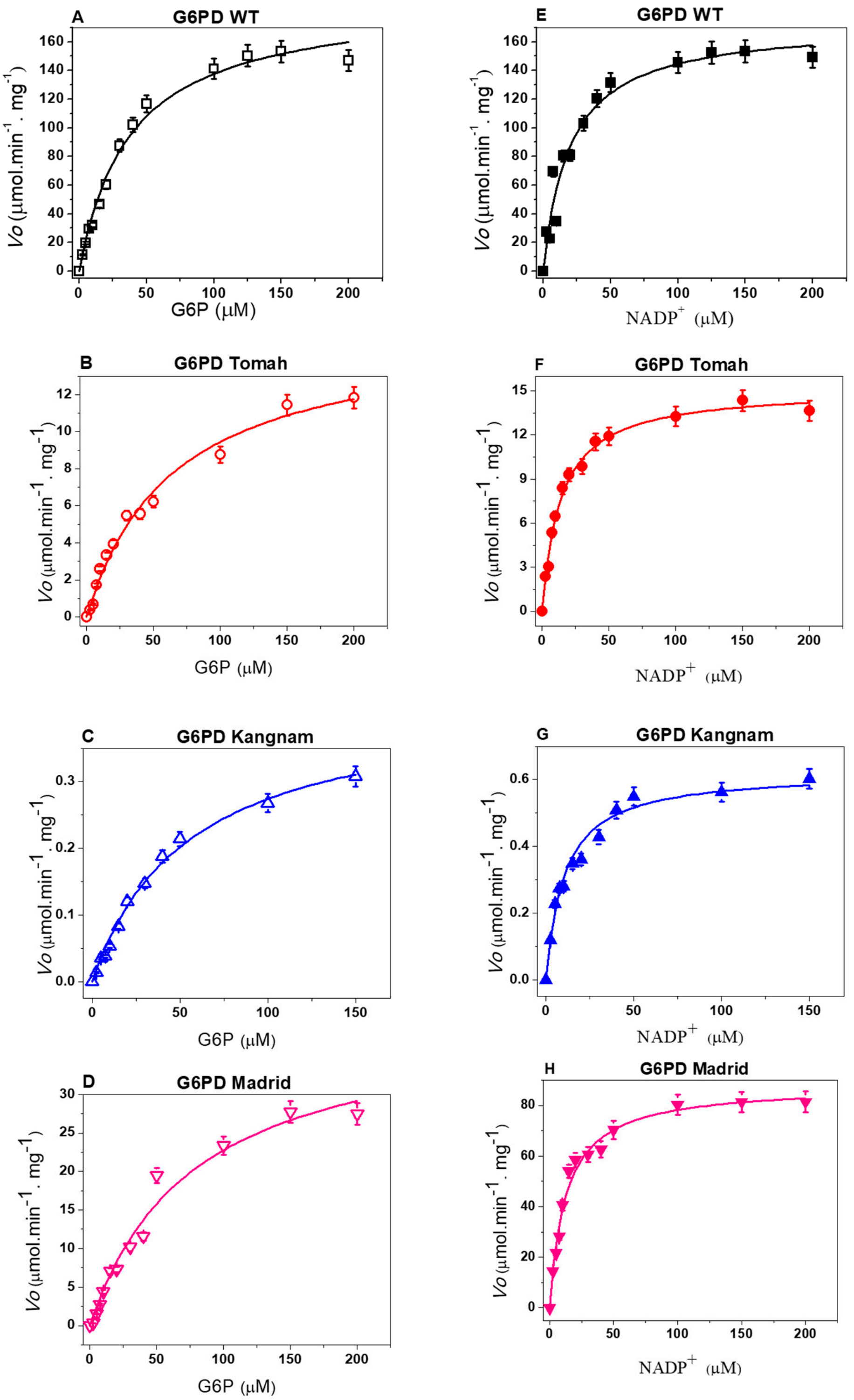

Figure S2. Kinetic assays of WT-G6PD (A, B) and the three G6PD variants: (C, D) G6PD Tomah (C385R), (E, F) G6PD Kangnam (C385G), and (G,H) G6PD Madrid (C385W). The initial velocities (*Vo*) of each concentration of the physiological substrates of G6PD (G6P and $NADP^+$) were fitted to the Michaelis–Menten equation via nonlinear regression calculations. The data represent means ± SDs from three independent experiments.

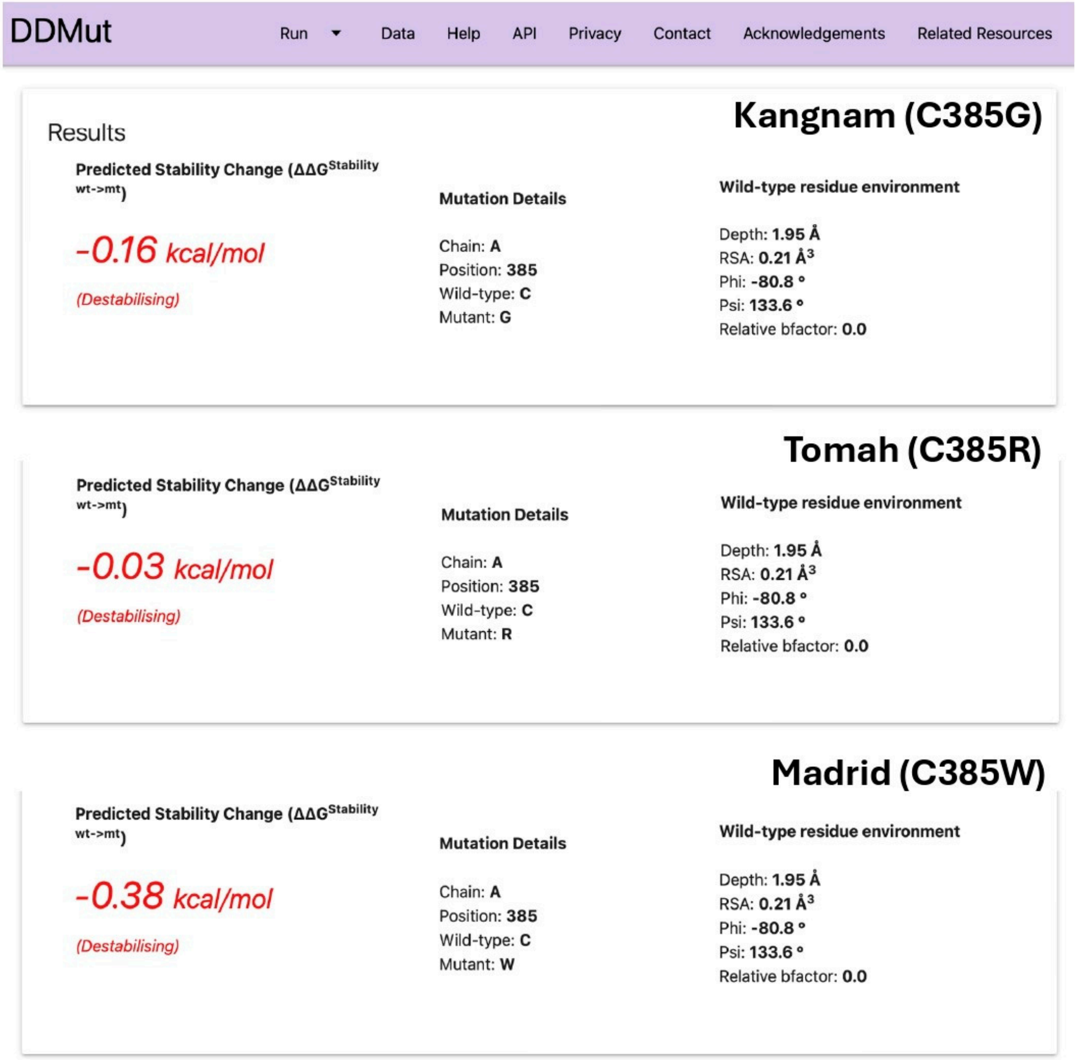

Figure S3. DDMut single position analysis on G6PD C385 variants. A single structure of WT G6PD was analyzed with DDMut (https://biosig.lab.uq.edu.au/ddmut/, accessed October 27, 2024), a deep-learning model that integrated a graph-based representation of the localized 3D environment of C385. This approach gave a small destabilizing effect associated with each mutation, and predicted changes in volume around C385. DDMut achieved pearson's correlation of up to 0.70 (RMSE: 1.37 kcal/mol) on predicting single point mutation on cross-validation [43].

Table S1. *In-silico* analysis of missense mutations of the *G6PD* gene reported in exon 10.

	Tomah	Kangnam	Madrid
Protein change	C385R	C385G	C385W
WHO category	Class I	Class I	Class I
PolyPhen-2	Bening<br>0.028	Bening<br>0.076	Probably Damaging<br>0.994
MutPRed	0.772<br>Loss of allosteric site at N388	0.725<br>Loss of Helix<br>Gain of Loop<br>Loss of Allosteric site at N388<br>Gain of acetylation at K386	0.777<br>Loss of Allosteric site at N388
Align GVGD*	Class C65, GD (179.53)	Class C65, GD (158.23)	Class C65, GD (214.36)

*Align GVGD: The prediction classes form a spectrum (C0, C15, C25, C35, C45, C55, C65) with C65 most likely to interfere with function and C0 least likely.
